# Supplementary material for: Decapping protein EDC4 regulates DNA repair and phenocopies BRCA1
Source: Nat Commun. 2018 Mar 6;9:967. doi: 10.1038/s41467-018-03433-3 (PMC5840268; doi:10.1038/s41467-018-03433-3)
Supplement: Supplementary file 1 — Supplementary Information [file 41467_2018_3433_MOESM1_ESM.pdf]

Decapping protein EDC4 regulates DNA repair and phenocopies BRCA1

Hernández et al.

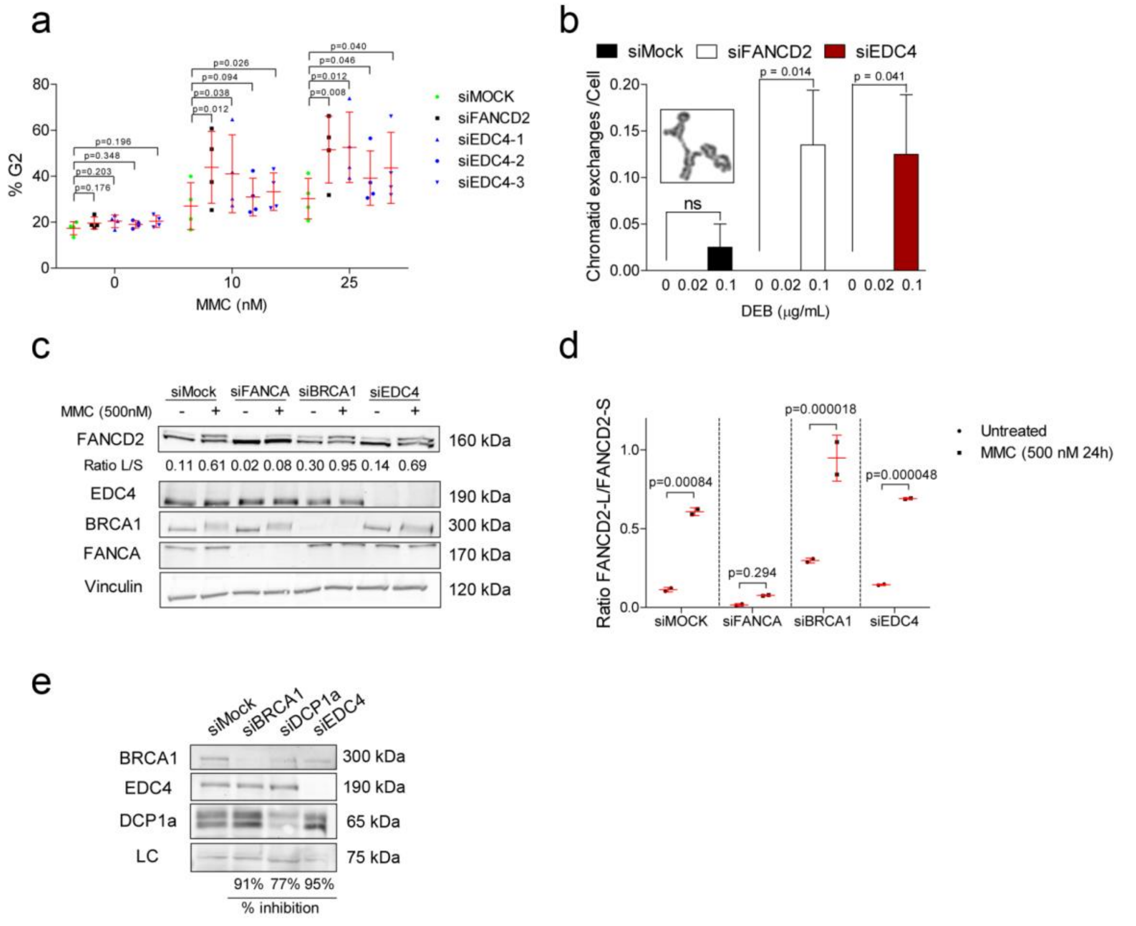

Supplementary Figure 1. a, EDC4 depletion causes G2/M cell cycle arrest after MMC exposure in HeLa cells. Data shown represent results from four combined independent experiments. Means were statistically compared using the 2-tailed Student's t test. Error bars indicate mean ± s.d. **b**, Quantification of DEB-induced chromatid-type exchanges in EDC4 depleted primary fibroblasts. Data shown represent results from the analysis of 40 metaphases (two experiments of 20 metaphases each one). Error bars indicate mean ± s.e.m. The statistical test performed in each inhibition to compare chromatid exchange/cell (0 vs 0.1 μg/mL DEB) was a Mann-Whitney test. **c**, Immunoblots showing that EDC4 is dispensable for FANCD2 monoubiquitination after MMC exposure in FANCA, BRCA1 or EDC4 depleted HeLa cells. **d**, Quantification of the levels of FANCD2 monoubiquitination in FANCA, BRCA1 or EDC4 depleted HeLa cells. Data shown represent results from two combined independent experiments.

Means were statistically compared using the 2-tailed Student's t test. Error bars indicate mean ± s.d. **e**, Immunoblots showing the inhibition efficiency of DCP1a, EDC4 and BRCA1 in HeLa cells.

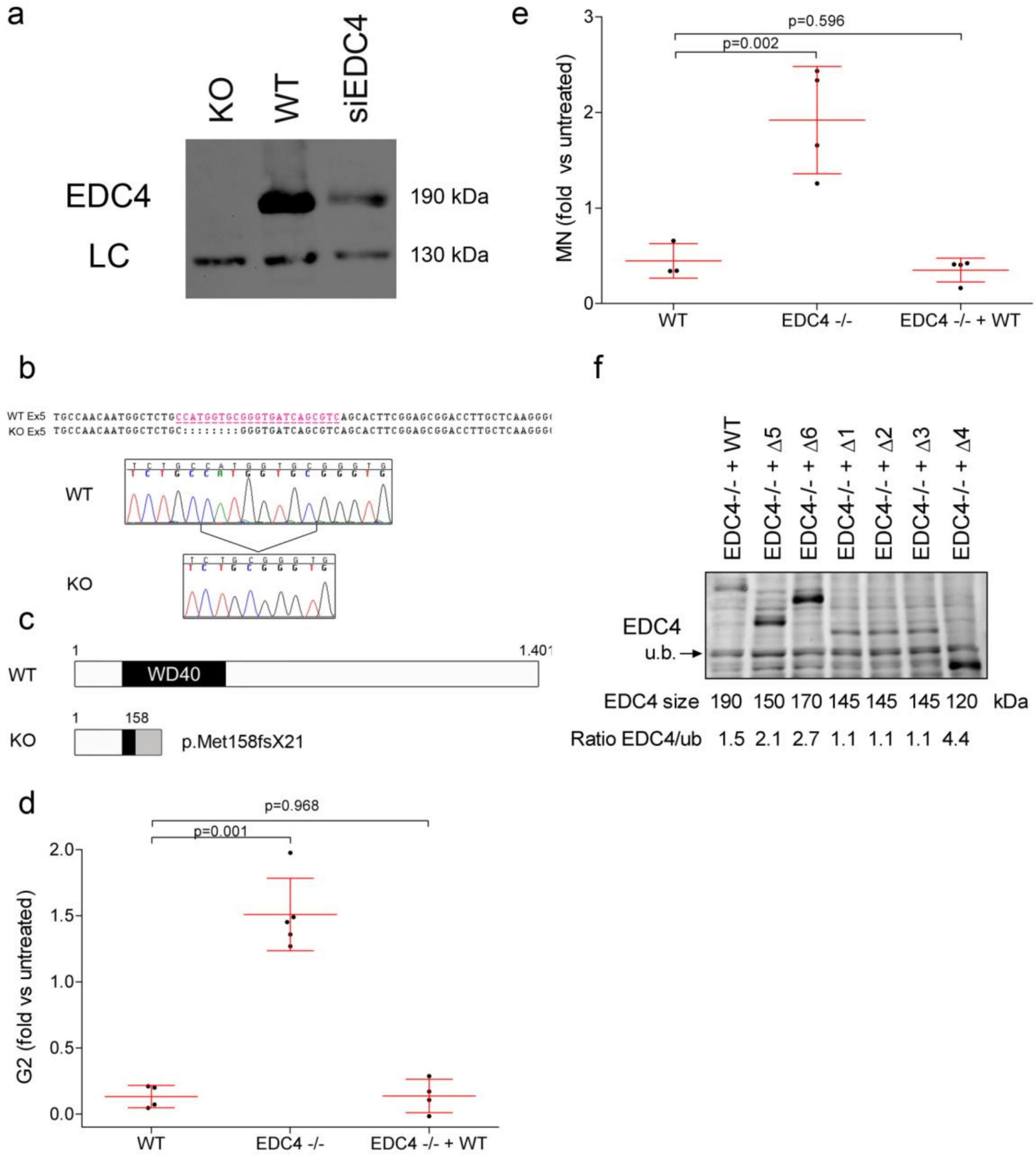

Supplementary Figure 2. a, Immunoblots showing an HEK293T EDC4 KO clone obtained by CRISPR/Cas9. siRNA against EDC4 was included as a control to identify the specific EDC4 band. **b**, Alignment of the Cas9 target sequence at exon 5 of EDC4. A homozygote 8bp deletion was found leading to a premature stop codon. **c**, Diagram comparing the WT form of EDC4 with the mutant protein translated from the CRISPR/Cas9 mutated allele. **d**, HEK293T EDC4-/- cells exhibit DEB-induced G2/M cell cycle arrest and **e**, DEB-induced chromosome fragility as measured by the flow

cytometric MN assay. Data shown in d-e represent results from at least three combined independent experiments. Means were statistically compared using one-way analysis of variance (ANOVA) followed by a Dunnett's multiple comparison test. Mean ± s.d. are shown. **f**, Immunoblots showing the levels of expression of HA-tagged EDC4 deletion mutants in HEK293T cells with an anti-HA antibody. Molecular weight is indicated at the bottom of the panel; u.b.: unspecific band that serves as internal loading control. Ratio between EDC4 and the unspecific band serves as a loading control. This experiment was performed twice, and we show a representative Western blot.

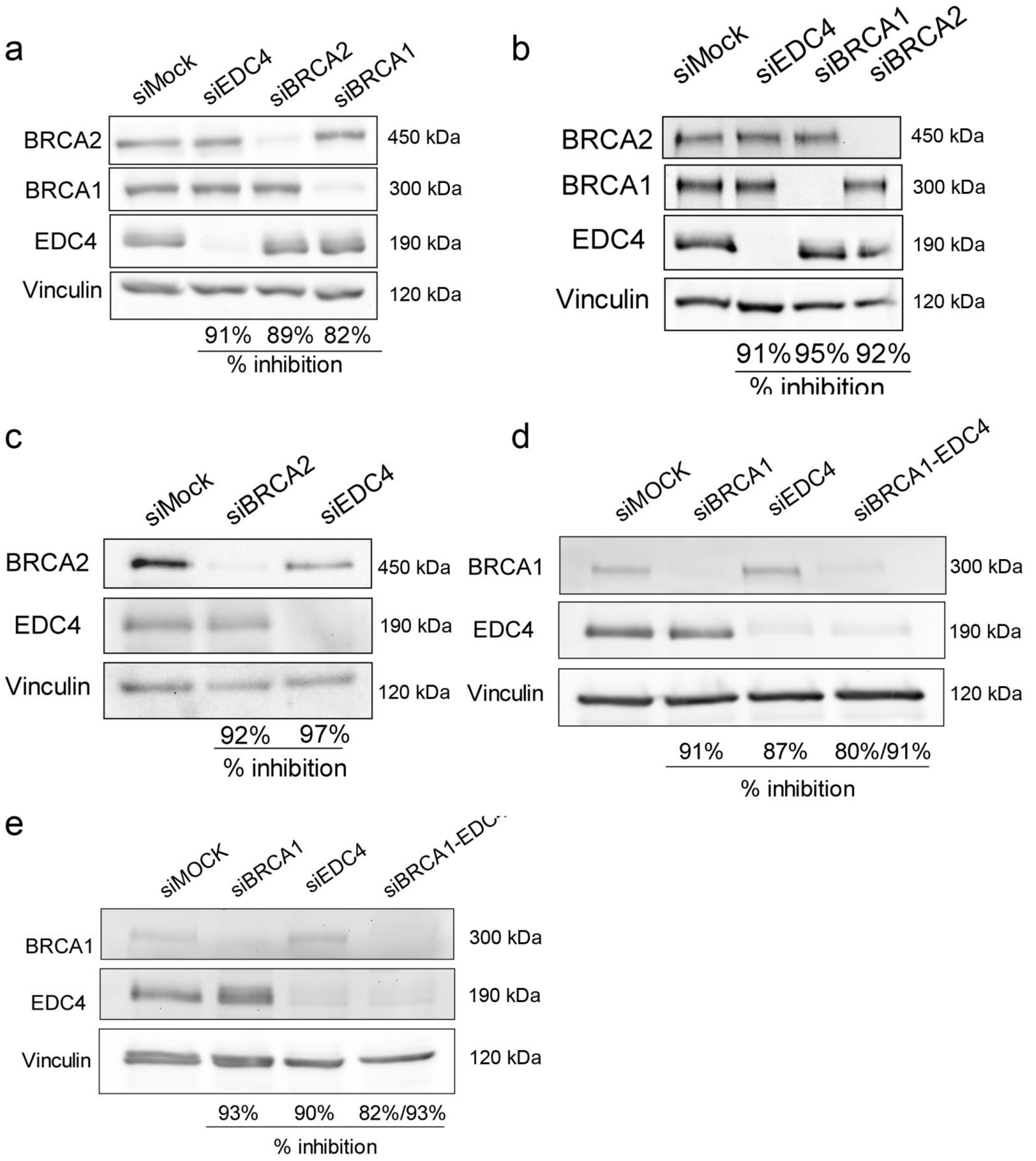

Supplementary Figure 3. a, Immunoblots showing the inhibition efficiency of EDC4, BRCA1 and BRCA2 in U2OS-DR-GFP cells by siRNA. **b**, Immunoblots showing the inhibition efficiency of EDC4, BRCA1 and BRCA2 in HeLa cells. **c**, Immunoblots showing the inhibition efficiency of EDC4 and BRCA2 in HeLa cells. **d**, Immunoblots showing the inhibition efficiency of EDC4, BRCA1 and the simultaneous inhibition of EDC4 and BRCA1 in HeLa cells. **e**, Immunoblots showing the inhibition efficiency of

EDC4, BRCA1 and the simultaneous inhibition of EDC4 and BRCA1 in U2OS-DR-GFP cells by siRNA.

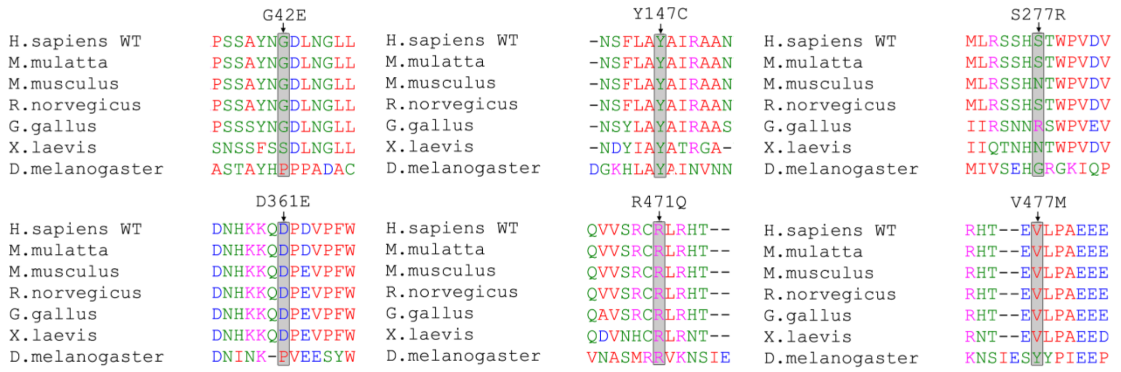

Supplementary Figure 4. Aminoacidic sequence alignment of the EDC4 from different species to show that residues found mutated in breast cancer patients are highly conserved.

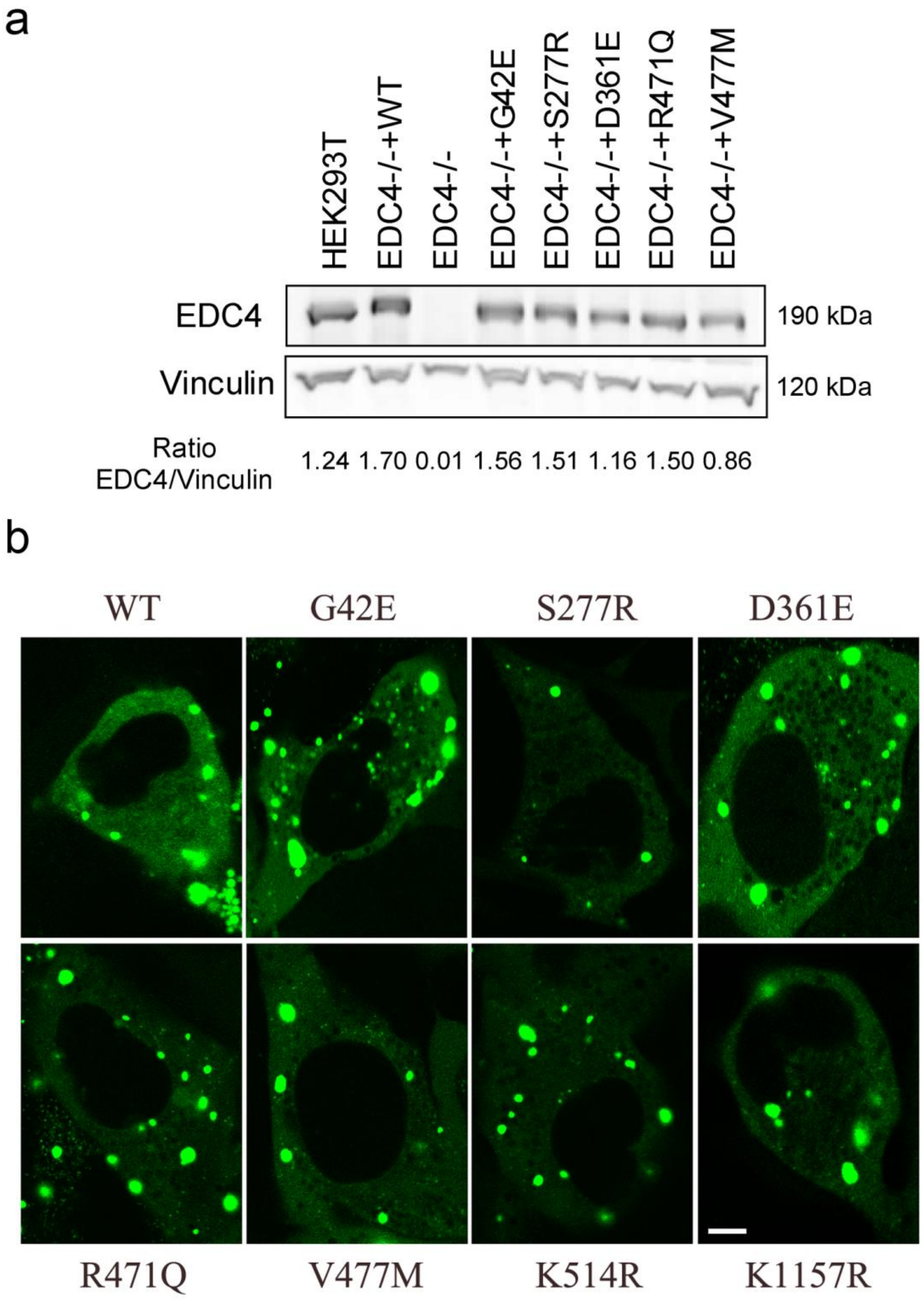

Supplementary Figure 5. a, Immunoblots showing EDC4 expression levels in EDC4 deficient HEK293T cells transduced with G42E, S277R, D361E, R471Q and V477M EDC4 mutants. This experiment was performed twice, and we show a representative Western blot. **b**, Confocal images of cells expressing GFP-fused forms of EDC4 WT,

missense mutations found in BRCAx patients and point mutations affecting lysines required for ICL-repair. Scale bar represents 10 µm.

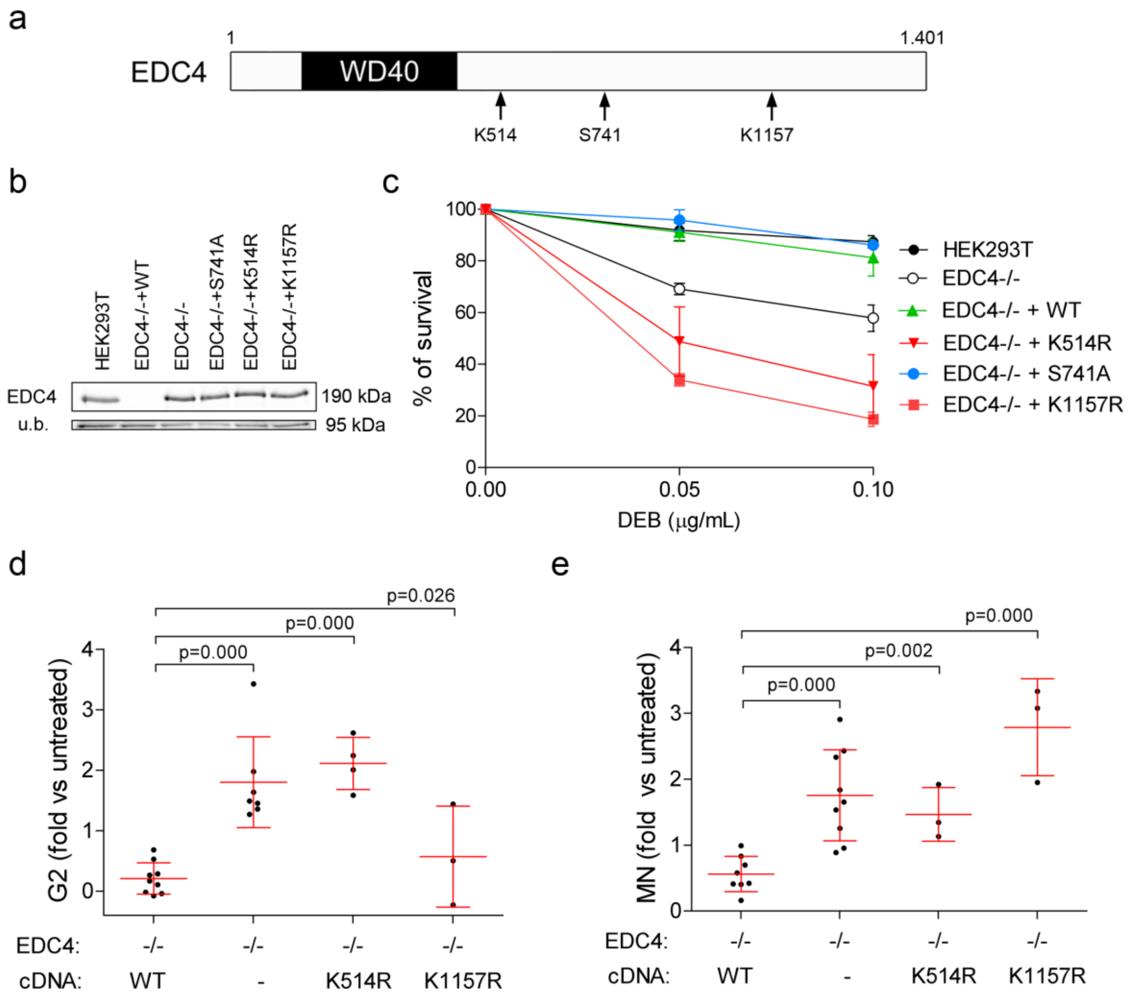

Supplementary Figure 6. **a**, Diagram showing the localization of EDC4 residues previously reported in proteomic screenings to be post-translationally modified in response to DNA damage. **b**, Immunoblots showing EDC4 expression levels of K514R, S741A and K1157R mutants in HEK293T EDC4 KO cells. u.b.: unspecific band that serves as internal loading control. **c**, Alteration of the K514 and K1157 residues, but not S741, results in a failure to revert DEB sensitivity of HEK293T EDC4-/- cell line. Means were statistically compared using the 2-tailed Student's t test. No statistical difference was observed between WT, S741A(p=0.214;p=0.393) and corrected cells (p=0.751;p=0.824) while the ubiquitination mutants where found statistically different compared with the WT cell line (K514R, p=0.001 and p=0.000; K1157R, p=0.000 and p=0.000). Data show the results of three combined experiments. **d**, **e**, K514R and

K1157R mutants show an increased G2/M cell cycle block and MN induction after DEB treatment. Means were statistically compared using one-way analysis of variance (ANOVA) followed by a Dunnett's multiple comparison test. Data shown in c-e represent results from at least three combined independent experiments in HEK293T cells. Error bars indicate mean ± s.d.

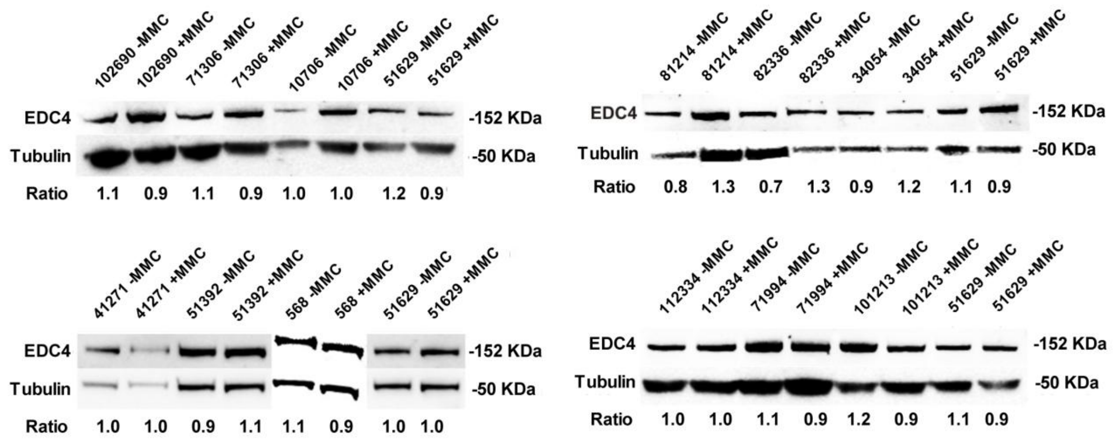

Supplementary Figure 7. Immunoblots showing that EDC4 is expressed in the studied FA patients. A EDC4/Tubulin ratio is included as a loading control.

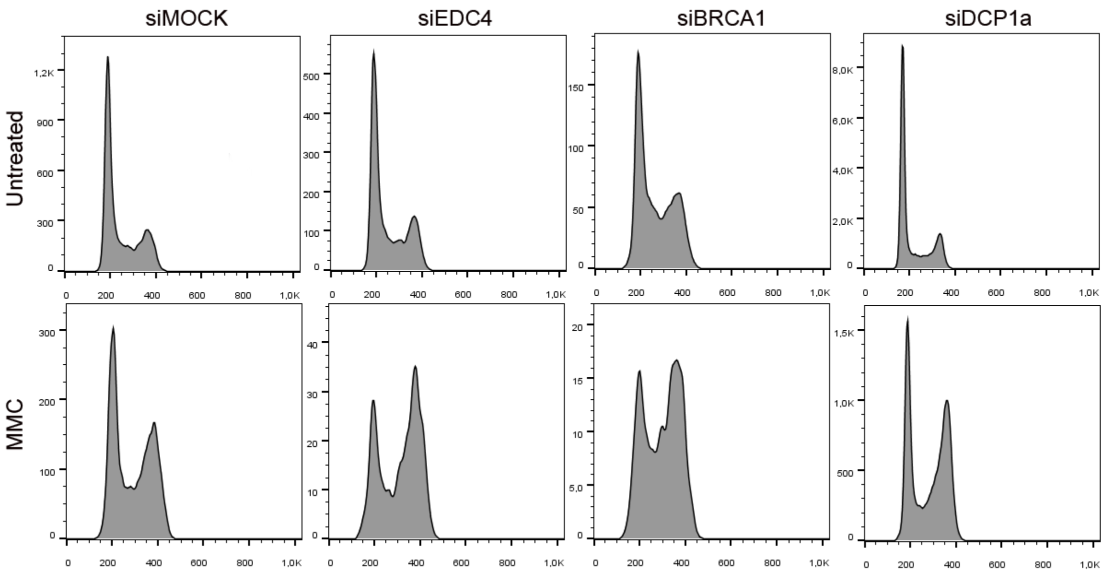

Supplementary Figure 8. Histograms showing the cell cycle distribution of Mock, EDC4, BRCA1 and DCP1a HeLa depleted cells and the effect of MMC treatment on the amount of cells arrested in the G2 phase of the cell cycle.

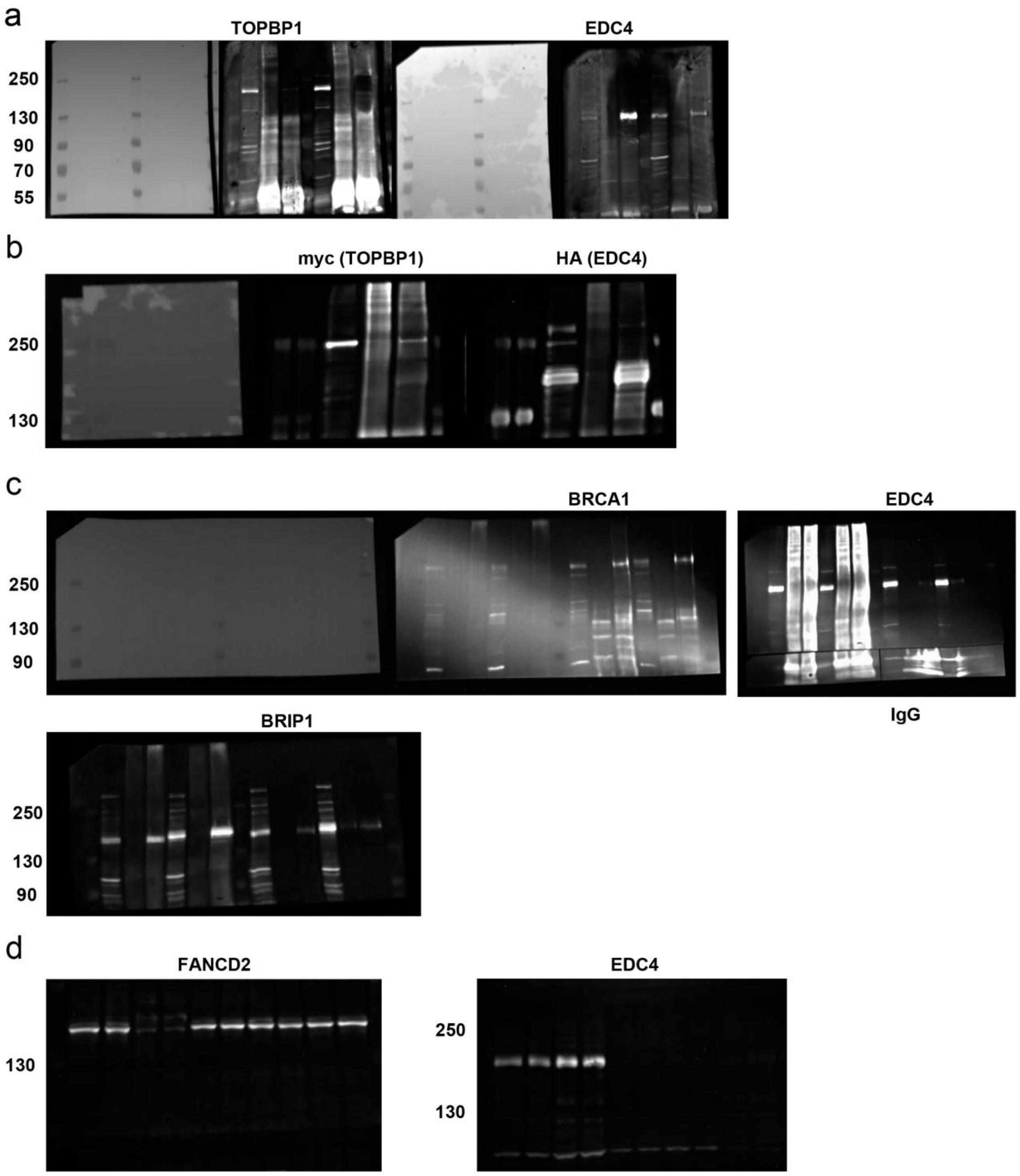

Supplementary Figure 9. Uncropped and unprocessed images from blots. **a**, Western blots of the IP shown in Figure 1b blotted against TOPBP1 and then EDC4. **b**, Western blots showing the results of the IP in Figure 1c blotted using antibodies against myc-tagged TOPBP1 and then detecting the HA-tagged EDC4. **c**, Western blots of the IP corresponding to Figure 1f. First BRCA1 was detected, then EDC4/IgG and then BRIP1. **d**, Western blots shown in Figure 2a assessing the inhibition of FANCD2 and EDC4.

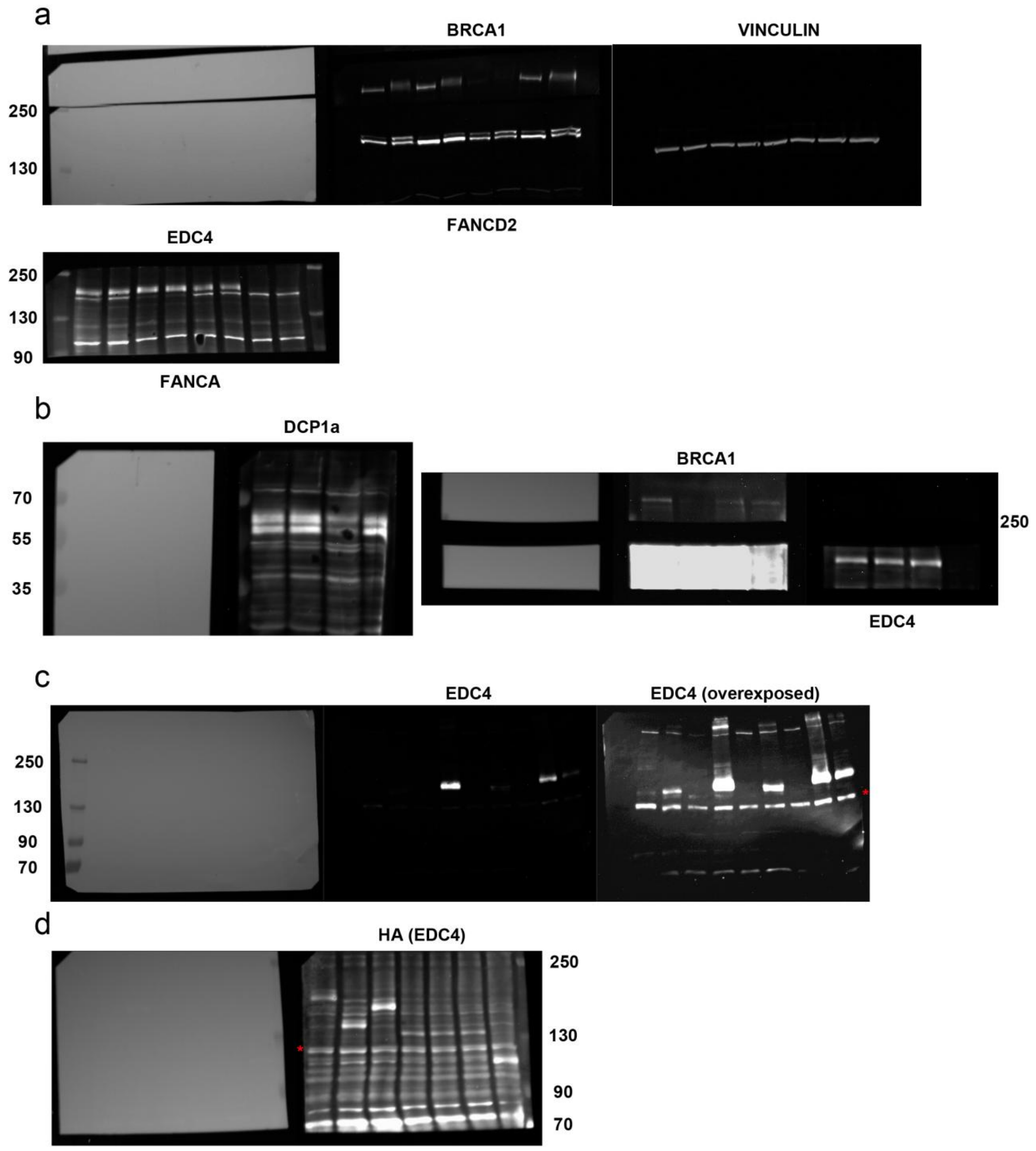

Supplementary Figure 10. Uncropped and unprocessed images from blots. **a**, Western blots of the FANCD2 monoubiquitination experiment shown in Supplementary Figure 1c. **b**, Images showing the inhibition of DCP1a, BRCA1 and EDC4 corresponding to Supplementary Figure 1e. **c**, Analysis of EDC4 expression of the CRISPR clones shown in Supplementary Figure 2a. Asterisk marks the band used as loading control. **d**, Analysis of EDC4 expression of the deletion mutants as shown in Supplementary Figure 2f. The WT and deletion mutants were detected using antibodies raised against the HA tag. Asterisk marks the band used as loading control.

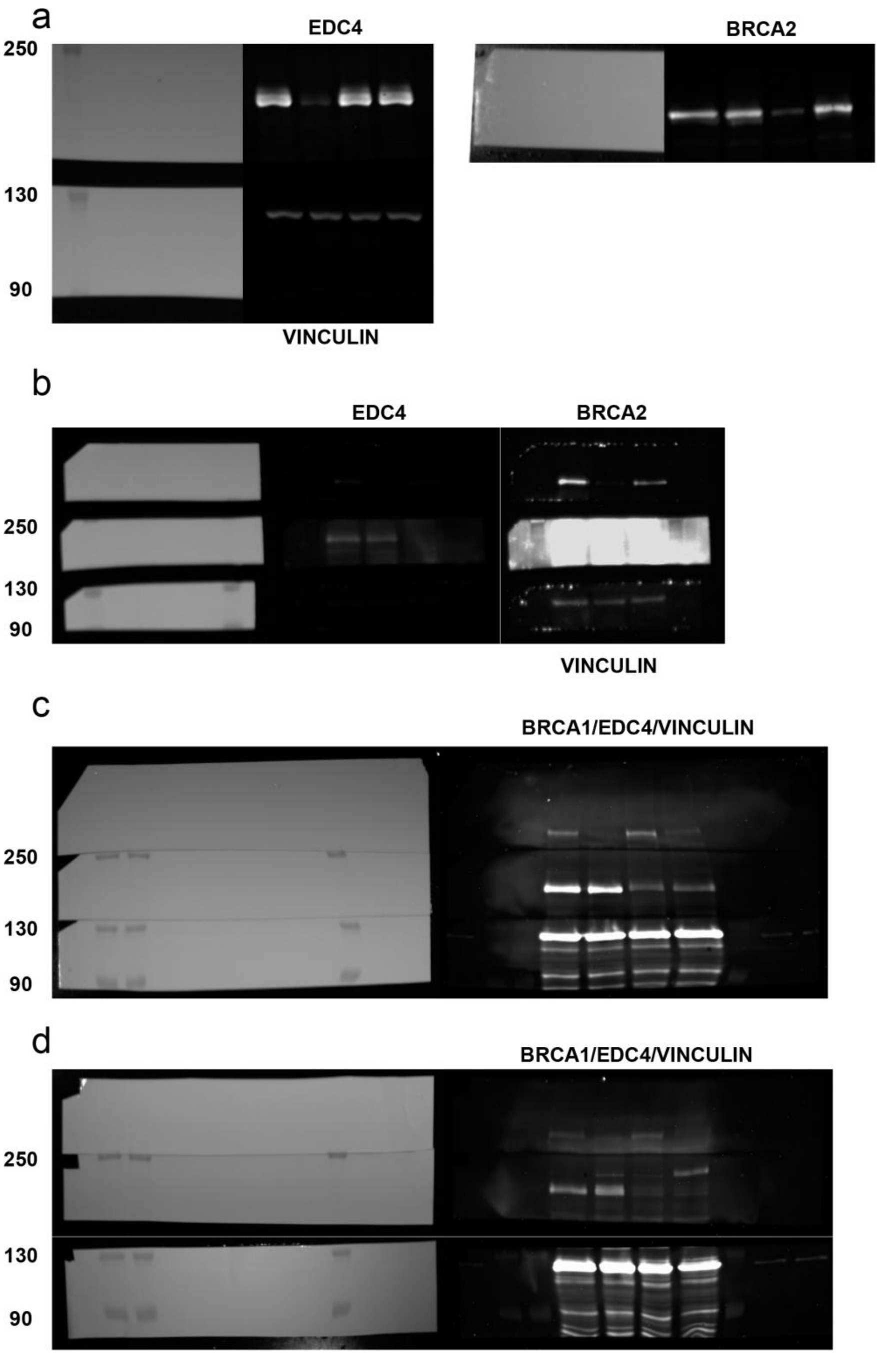

Supplementary Figure 11. Uncropped and unprocessed images from blots. **a**, Western blots of the inhibition efficiency of EDC4 and BRCA2 as shown in Supplementary Figure 3a. **b**, Blot of EDC4 and BRCA2 demonstrating the inhibition of the proteins according to Supplementary Figure 3c. **c**, Images representing the panel shown in Supplementary Figure 3d. **d**, Inmunoblots of the western blot shown in Supplementary Figure 3e

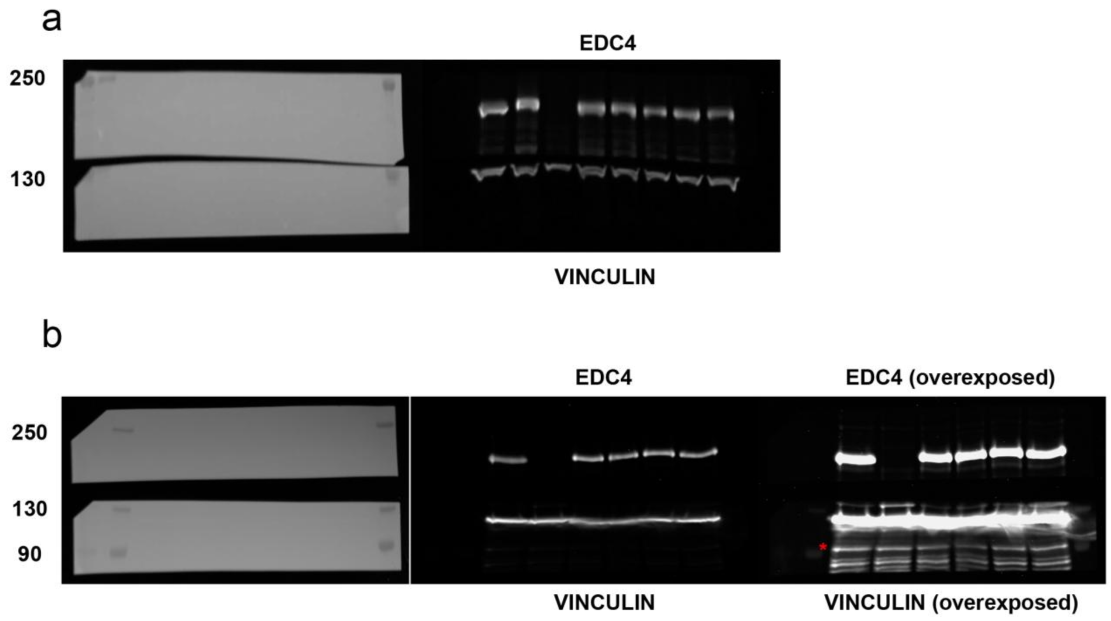

Supplementary Figure 12. Uncropped and unprocessed images from blots. **a**, Western blots showing the expression levels of EDC4 in KO cells infected with lentiviral particles to express the mutations found in breast cancer patients as shown in Supplementary Figure 5a. **b**, Immunoblots showing the expression of cells expressing point mutations affecting residues affected by post-translational modifications in EDC4 corresponding to the panel represented in Supplementary Figure 5b. Asterisk marks the band used as loading control.

Construct	Primers
G42E	ATCCAGTGCCTACAATGAGGACCTCAATGGACTTC
	GAAGTCCATTGAGGTCCTCATTGTAGGCACTGGAT
S277R	CGCTCCAGCCACCGTACCTGGCCTG
	CAGGCCAGGTACGGTGGCTGGAGCG
D361E	TCTGTGACAACCATAAGAAACAAGAACCTGATGTCCCTT
	AAGGGACATCAGGTTCTTGTTTCTTATGGTTGTCACAGA
R471Q	GTGAGTCGCTGCCAGCTACGGCACACT
	AGTGTGCCGTAGCTGGCAGCGACTCAC
V477M	CGGCACACTGAGATGCTGCCTGCCG
	CGGCAGGCAGCATCTCAGTGTGCCG
S741A	TTCCACTGCCCTGGCCCAGGACATCCC
	GGGATGTCCTGGGCCAGGGCAGTGGAA
K514R	CTCTTTTGTGTGCATACTAGGGCACTGCAAGATGTGCAG
	CTGCACATCTTGCAGTGCCCTAGTATGCACACAAAAGAG
K1157R	GCTAGAAAGCCACATGAGGAGCCGGAAGGC
	GCCTTCCGGCTCCTCATGTGGCTTTCTAGC
Δ1	GCCGCGATCGCCATGGATGTTAGCCAGATC
	GATCTGGCTAACATCCATGGCGATCGCGGC
Δ2	CAACCTGATTGCTGTGCACCCTGTGCTGAGCT
	AGCTCAGCACAGGGTGCACAGCAATCAGGTTG
Δ3	ACCTGGCCTGTGGATGTTGCCTTCATGACACC
	GGTGTCATGAAGGCAACATCCACAGGCCAGGT
Δ4 (Fragment 1)	ATAGCGATCGCCATGGCCTC
	TAGGTGTCATGAAGGCAACATCCACAGGCCAG
Δ4 (Fragment 2)	TGGCCTGTGGATGTTGCCTTCATGACACCTAG
	GCTACGCGTAGGGAGGCTGG
Δ5	TCAAGTCCAAGAACTTGACTGATACGCGTACGCGG
	CCGCGTACGCGTATCAGTCAAGTTCTTGGACTTGA
Δ6	CCTCAATCAGGCCACGCGTACGCGGC
	GCCGCGTACGCGTGGCCTGATTGAGG

Supplementary Table 1. List of primers used for generation of different EDC4 mutant forms by site-directed mutagenesis.

Patient ID	Non-pathogenic variants found
**41271**	c.747T>C ; g.4592T>C ; g.9153_9155 delTCT ***(rs1175339386)***
**60749**	c.2876G>A ***p.R959K***
**51392**	c.2876G>A ***p.R959K***
**81214**	c.2462C>T p.T821I ; c.2463T>A
**71994**	c.202A>G p.T68A ; g.3094G>A ; g.3097G>A ; g.10160G>A
**82336**	c.2876G>A ***p.R959K***
**11347**	c.2876G>A ***p.R959K*** ; c.4113G>A ; g.11033G>A ***(rs34607252)***
**644**	c.2876G>A ***p.R959K*** ; c.2926T>G ***p.C976G***
**10706**	-
**357/ 1027**	-
**569/1/1978**	-
**858/2/2716**	g.6372C>T ***(rs76483578)*** ; c.2926T>G ***p.C976G***
**34054**	-
**102690**	-
**123104**	-

Supplementary Table 2. Table with the non-pathogenic variants found in the unassigned Fanconi downstream patients analyzed.
